# Correlation between surgical segment mobility and paravertebral muscle fatty infiltration of upper adjacent segment in single-segment LDD patients: retrospective study at a minimum 2 years’ follow-up

**DOI:** 10.1186/s12891-023-06137-y

**Published:** 2023-01-12

**Authors:** Jianbin Guan, Dingyan Zhao, Tao Liu, Xing Yu, Ningning Feng, Guozheng Jiang, Wenhao Li, Kaitan Yang, He Zhao, Yongdong Yang

**Affiliations:** Dongzhimen Hospital Beijing University of Chinese Medicine, Haiyuncang No.5, Dongcheng District 100700 Beijing, China

**Keywords:** Isobar, Posterior pedicle screw fixation, Posterolateral intertransverse lumbar fusion, Lumbar degenerative disease, Paraspinal muscles fat infiltration

## Abstract

**Objective:**

The purpose of this study was to investigate the relationship between surgical segment mobility and fatty infiltration of the adjacent segment paravertebral muscles in patients with single-segment lumbar degenerative disease (LDD) who underwent decompression with fusion or dynamic stabilization.

**Methods:**

Retrospective analysis of patients who underwent lumbar decompression combined with titanium rod fixation intertransverse fusion (PITF group), Isobar TTL dynamic stabilization (TTL group) or Isobar EVO dynamic stabilization (EVO group) for single-segment lumbar degenerative disease, from March 2012 to July 2018. The preoperative and final follow-up clinical indexes C-LDSI and the measured imaging indexes (range of motion of the surgical segment and the upper adjacent segment, and Goutallier grade of the upper adjacent segment) were counted, and the differences between the preoperative and final follow-up indexes were compared.

**Results:**

According to the inclusion and exclusion criteria, 68 patients were included in this study, 21 in the PITF group, 24 in the TTL group, and 23 in the EVO group. At the final follow-up, the C-LSDI score had significantly higher in the PITF group than the TTL and EVO groups, and the C-LSDI score was a very strongly negatively correlated with ROM of surgical segment (*r*=-0.7968, *p* < 0.001). There was a strong negative correlation between surgical segment and upper adjacent segment mobility (*r* = -0.6959, *p* < 0.001). And there was a very strong negative correlation between ROM of surgical segment and upper adjacent segment paravertebral muscle Goutallier classification (*r* = -0.8092, *p* < 0.001), whereas the ROM of the upper adjacent segment was strong positive correlated with the Goutallier classification (*r* = 0.6703, *P* < 0.001).

**Conclusion:**

Compared with decompression combined with rigid fusion, decompression combined with dynamic fixation for single-segment lumbar degenerative disease can significantly reduce postoperative low back stiffness. And a certain range of increased mobility of the dynamic stabilization device can effectively reduce the compensatory mobility of the upper adjacent segment and slow down the fatty infiltration of the paravertebral muscle in the adjacent segment.

## Introduction

Although posterior lumbar fusion with pedicle screws is a “gold-standard” for the treatment of lumbar degenerative diseases, the long-term effects of rigid fixed fusion accelerate the degeneration of adjacent segments, such as adjacent segment instability, spinal stenosis, disc degeneration, fracture, and other long-term degenerative manifestations [1–3]. After intervertebral fusion, the increased range of motion (ROM) and abnormal stresses in adjacent segments cause abnormalities in one or more structures, including vertebrae, disc, facet joint, peripheral ligament, and paravertebral muscle, resulting in adjacent segment degeneration (ASD) [4, 5]. As a result, a variety of mobility-preserving devices and surgical approaches, such as non-fusion and hybrid, have gradually emerged clinically to reduce interference with the biomechanical environment of spine in order to prevent or slow down the progression of ASD [6, 7].

In recent years, clinical practitioners have paid attention to the role of the paravertebral muscles in the management and prognosis of the lumbar and cervical spines, and paravertebral muscle degeneration is gradually being recognized as an important cause of ASD [8, 9], and fatty infiltration of the paravertebral muscles is closely associated with degeneration of other structures of the spine [10–14]. Onesti et al [15]. found that the fatty infiltration of the paravertebral muscles that occurs after spinal fusion was found to cause failed back syndrome, suggesting an important association between persistent and recurrent low back pain after lumbar spine surgery and fatty infiltration of the paravertebral muscles. In addition, postoperative stiffness of the low back is a common problem after lumbar spine surgery [16]. Although numerous predisposing factors are known, such as ischemia and paravertebral muscle denervation caused by long incisions, extensive dissection, and long intraoperative muscle strains [17–20]. However, it is now thought that abnormal mobility and stress transmission after fusion causes paravertebral muscle overload, which is the primary cause of persistent, recurring postoperative pain and postoperative stiffness [21–23]. When the ROM of fixed segments is lost, muscle wasting atrophy and fatty infiltration of muscle tissue occur during the healing process [24], causing an increase in compensatory mobility of the adjacent segment and fatty infiltration of the paravertebral muscle of the adjacent vertebrae caused by stresses on the adjacent segment's paravertebral muscle exceeding its limits. Lin et al [25]. compared the effects of dynamic fixation systems and fusion on the paravertebral muscles of adjacent segment and discovered that patients undergoing posterior interbody fusion had higher grades of paravertebral muscle atrophy and fatty infiltration than patients undergoing posterior dynamic internal fixation. However, in this study, the comparison of unilateral K-rod dynamic fixation and posterior interbody fusion was more interference factors in investigating the differences in the effects of dynamic stabilization versus fusion on the adjacent segmental paravertebral muscles. In addition, whether there is a correlation between the mobility of dynamic fixation and fatty infiltration of the paravertebral muscles has not been reported in the literature.

In this study, we retrospectively analyzed the correlation between postoperative surgical segmental mobility and fatty infiltration of the upper adjacent segmental paravertebral muscles in patients with single-segment lumbar degenerative disease treated with lumbar decompression combined with titanium rod fixation for intertransverse fusion, combined with Isobar TTL dynamic fixation or combined with Isobar EOV dynamic fixation.

## Methods

### Patients and methods

This study retrospectively reviewed 189 consecutive patients with single segment lumbar degeneration diseases who were undergoing surgical treatment with PTIF or Isobar TTL/EVO between March 2012 and July 2018. According to the inclusion and exclusion criteria, 68 patients were included in this study, 21 in the PITF group, 24 in the TTL group, and 23 in the EVO group This study has been approved by the Ethical Committee of the Dongzhimen Hospital affiliated to Beijing University of Chinese Medicine (2022DZMEC-085–04). And the study was retrospective and patient records were anonymized and de-identified prior to analysis, no patient informed consent was available.

### Inclusion and exclusion criteria

The inclusion criteria were as follows: (a) spinal surgery patient at the Dongzhimen Hospital affiliated to Beijing University of Chinese Medicine from March 2012 and July 2018; (b) patients with lumbar degenerative disease in single segment (L4/5 or L5/S1) who were undergoing surgical treatment with PTIF or Isobar TTL/EVO; (c) surgical segment Pfirrmann grading [26]  ≥ III and upper adjacent segment disc Pfirrmann grading < II; (d) surgical segment and upper adjacent segment Modic changes [27] type 1 and 2 (All patients included in this study were Modic changes type 1 and 2, which was mainly to reduce the effect of Modic change on the intervertebral disc and paravertebral muscles); (e) spondylolisthesis degree I for Isobar TTL/EVO group and spondylolisthesis degree II for PITF group (In order to reduce the interference of lumbar spondylolisthesis grades with lumbar mobility and paravertebral muscles, we excluded patients with more than degree II lumbar spondylolisthesis). The surgical segment of L4/5 and L5/S1 were chosen because the paravertebral muscle has the largest area at L3/L4 and L4/L5 [8, 28–30]; (f) follow-up for at least for 24 months.

The exclusion criteria were as follows: (a) upper adjacent segment disc Pfirrmann grading > II; (b) upper adjacent segment facet joint Fujiwara classification [31] ≥ grade 3; (c) upper adjacent segment Goutallier classification > grade 2; (d) lumbar spondylolysis; (e) severe scoliosis or sagittal or coronal imbalance; (f) serious internal disease, severe osteoporosis, anxiety, depression or other psychological disorders. The flow chart of case screening for each group is as follows.

The flow chart of case screening for each group.

### Internal fixation devices

Titanium rod manufactured by Weigao in Shangdong China.

**Fig. 1:**
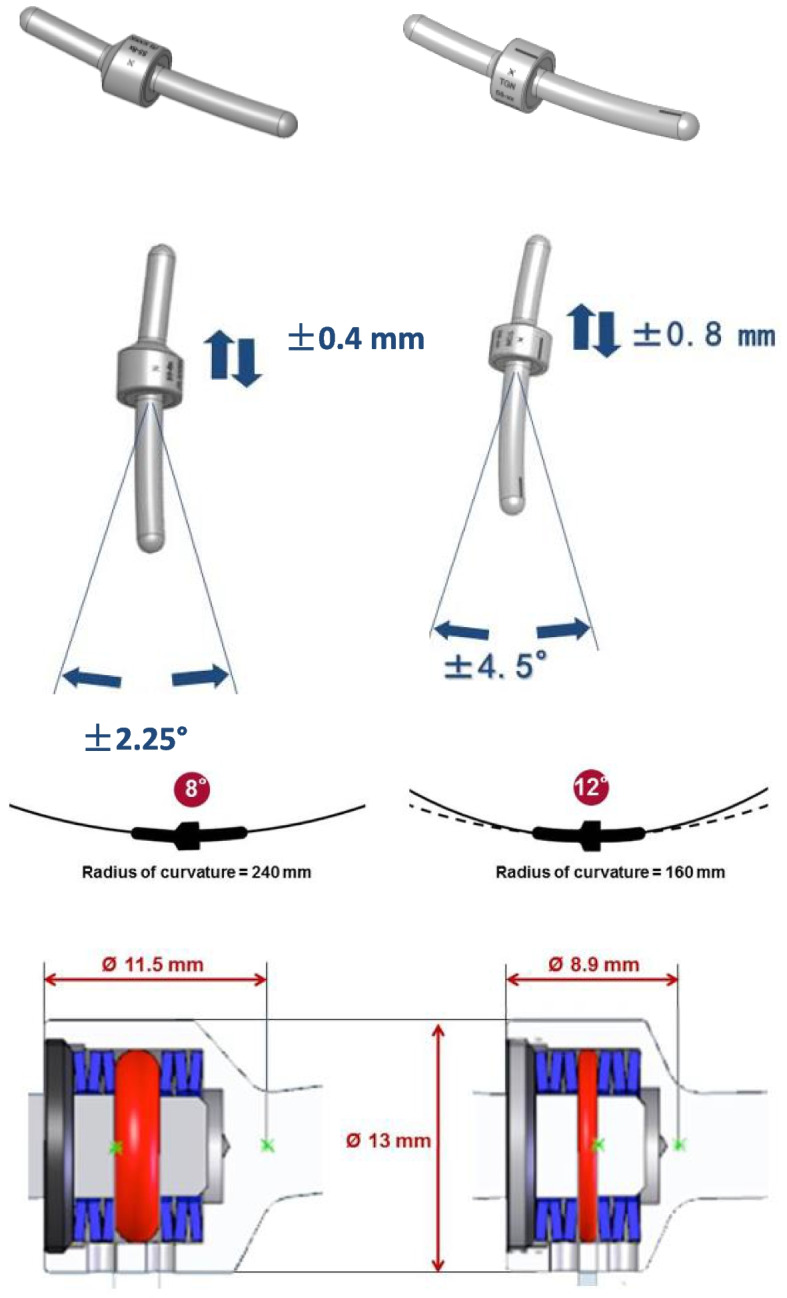
Isobar dynamic stabilization devices (right: Isobar TTL, left: Isobar EVO)

The Isobar TTL and EVO dynamic fixation devices manufactured by Scient'x-Alphatec in France. The difference between the TTL and the EVO we have described in detail in our previous articles [32]. (Fig. 1).

### Surgical procedure

The surgical procedure in the TTL and EVO group we had described in detail in our previous articles [32]. In PITF group, autologous bone is implanted between the transverse processes on both sides of the lesioned segment and the screw cap is tightened. In order to prevent infection, antibiotics were routinely given 24 h after surgery. Depending on the amount of drainage, the drainage tube was removed 24–48 h later. Three to five days following surgery, patients should continue wearing the brace to aid in getting out of bed while gradually retraining their lower back muscles. And in dynamic group, the brace is typically worn for one month following surgery. In the PITF group, the brace is typically worn for three month following surgery. After the brace is taken off, the patient is given functional exercise suggestions and told to regularly work out their low back.

### Clinical and radiological outcomes

 Clinical and radiological data were obtained before surgery and at the final follow-up. China lumbar stiffness disability index(C-LSDI) [33] were used to evaluate the lumbar stiffness of each patient. (Table1).

**Table 1:** China lumbar stiffness disability index

1. Bend to your feet to put on your underwear and pants on your own
2. Bend to your feet to put on your socks and shoes on your own
3. Bend forward to pick up a small object on the floor on your own
4. Bend forward to wash your face and hair without getting wet
5. Bathe the lower half of your body on your own
6. Perform personal hygiene functions following toileting
7. Bend forward to clean the floor on your own
8. Sit down on the chair and get up on your own
9. Lie down on the bed and get up on your own
10. Get in and out of a car on your own
11. Take a small object sideways on your own
**Response options and score for each item**
0 No effect at all
1 Help oneself but have minor effect
2 Help oneself but have effect
3 Help oneself but have significant effect
4 Cannot help oneself require assistance
5 Cannot do at all

**Fig. 2:**
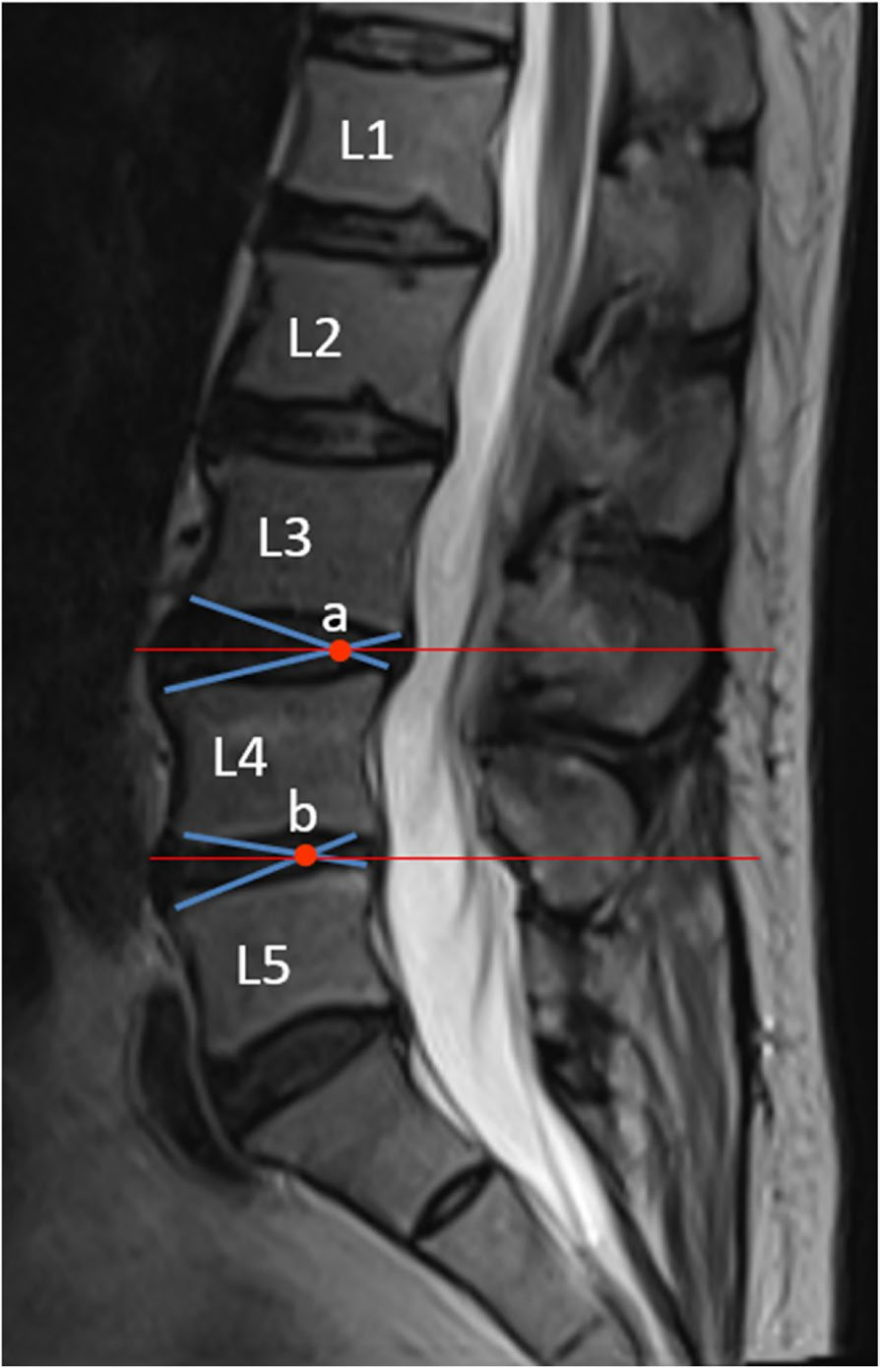
T2wI median sagittal correlation of the location of the axial paraspinal muscle measurements

**Fig. 3:**
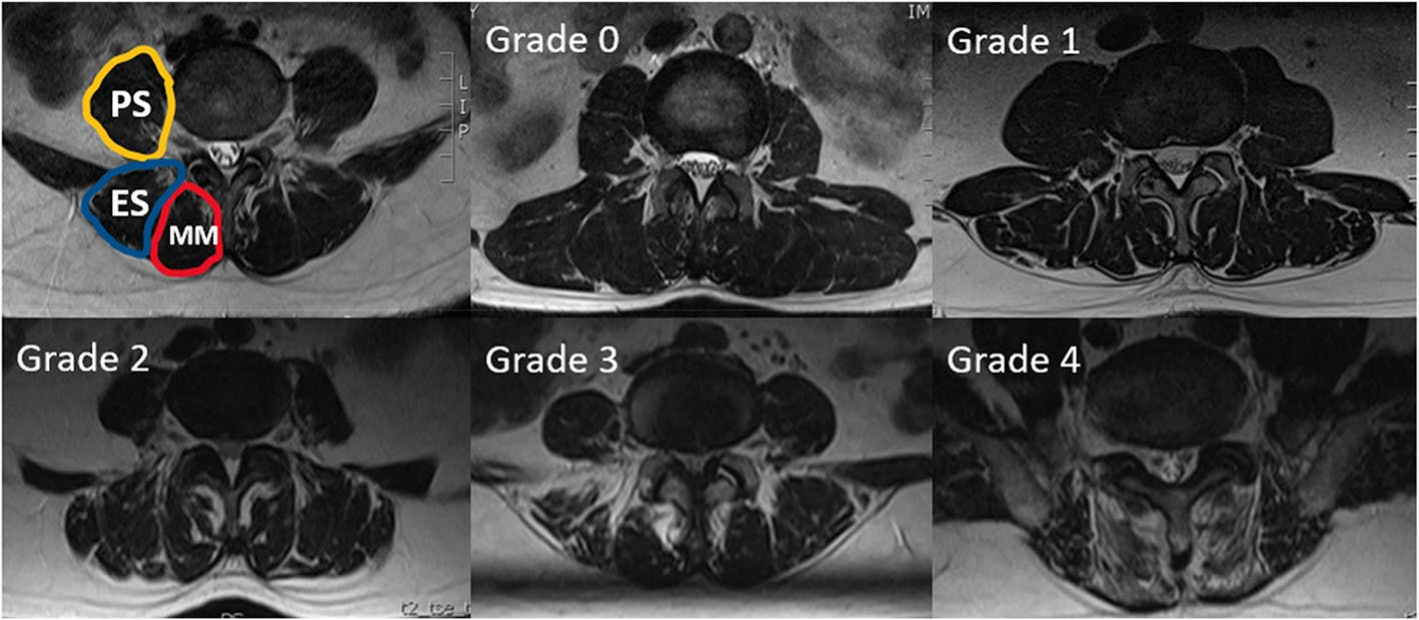
Goutallier classification. Every grade was defined as follows: grade 0, no intramuscular fat; grade 1, some fatty streaks present; grade 2, fat evident, but less than muscle tissue; grade 3, amounts of fat equal to amount of muscle; grade 4, more fat than muscle tissue. MM: Multifidus Muscle (Red) ES: Erector Spinae (Blue) PS: Psoas (Yellow)

Radiological outcomes were evaluated using the following: (a) the segmental ROM, which was calculated as the angle between the inferior surface of the upper vertebrae and the superior surface of the lower vertebrae on the lateral lumbar flexion–extension X-ray taken with the patient standing; (b) the paravertebral muscle is positioned axially through the median sagittal position, the anterior and posterior feet of the inferior border of the upper vertebral body and the superior border of the lower cone of the vertebral space, and the four points are crossed and intersected at a point, and a horizontal line is made through the point, which is the observed axial paravertebral muscle position (Fig. 2); (c)Goutallier grade, in which patients were considered to have Goutallier Grade 0 degeneration if there was no fat infiltration, Goutallier Grade 1 if fatty streaks were present, Goutallier Grade 2 if there was more muscle than fat, Goutallier Grade 3 if fat and muscle were present in equal quantity, and Goutallier Grade 4 if more fat was present than muscle [34, 35] (Fig. 3). The MRI system was a 1.5 Tesla Imaging System T (Signa HDxt 1.5 T GE, USA). Cross-sectional views of lumbar vertebrae were obtained using a fast spin-echo sequence system for T2WI. The slice width was 4 mm and the inter-slice gap was 1 mm. The acquisition matrix was 320/224. The sequence parameters were a repetition time of 3000 ms and an echo time of 102 ms for T2WI. The measurement of paraspinal fat infiltration acreage: the MRI T2 axial map of the same segment and the same level before and after the operation were selected. Image J software was applied to outline the periphery of the paraspinal muscles on the image with 4X magnification and the region is fat (Fig. 10).

The paravertebral muscles in this study were defined as ES + MM.

### Statistical analysis

Continuous variables were expressed as the mean ± standard deviation and were analyzed using SPSS 22.0 software (SPSS Inc., Chicago, IL, USA). We used one-way ANOVA for the statistical analysis to compare all means between groups, and we used a p value less than 0.05 to determine statistical significance. Categorical variables were compared using Fisher’s exact test. To assess the correlation between ROM (surgical segment or Upper segment) and Goutallier Grade of paraspinal muscles, we performed Pearson correlation coefficient or Spearman’s rank correlation coefficient. Correlations > 0.7 were considered very strong; 0.5–0.7, strong; 0.3–0.5, moderate; and < 0.3, weak.

## Results

According to the inclusion and exclusion criteria, 68 patients (27 males and 39 females, mean age 50.63 ± 8.32 years) **were** eventually included, with a mean follow-up time of 37.0 ± 19.97 months (24–57 months), of which the number of patients with PITF, Isobar TTL, and Isobar EVO were 21, 24, and 23, respectively. 51 patients operated on L4/L5 segment, and 16 patients operated on L5/S1 segment. There were no significant differences in age, gender, BMI, disease type, or postoperative lumbar anterior convexity angle between the three groups (P > 0.05), and the PITF group had a longer operative time and more intraoperative bleeding than the TTL and EVO groups (*P* < 0.05), but there were no significant differences between the TTL and EVO groups (P > 0.05) (Table 2).

**Table 2:** Patient demographics

	**PITF(*n* = 21)**	**TTL(*n* = 24)**	**EVO(*n* = 23)**	***P1***	***P2***	***P3***
**Pre-operation**						
Mean age, years	52.90 ± 5.94	50.16 ± 9.67	49.04 ± 8.55	0.514	0.278	0.888
F/M gender	9/12	10/14	8/15	0.861*		
Follow-up (Month)	39.62 ± 10.36	34.75 ± 10.31	37.00 ± 9.04	0.234	0.657	0.718
BMI in kg/m^2^, mean	24.99 ± 2.61	25.45 ± 2.19	25.64 ± 2.08	0.784	0.619	0.957
surgical segment L4–5/L5–S1	15/6	19/5	18/5	0.823*		
Disorder				0.856*		
Disc herniation	3	5	6			
Spinal stenosis	14	13	13			
Spondylolisthesis	4	6	4			
C-LSDI	9.42 ± 3.52	9.45 ± 3.69	9.13 ± 2.78	0.978	0.756	0.734
**Post-operation**						
Goutallier Grade of upper segment (no. of levels)				< 0.001^#^		
Grade 0	0	0	10(43.5%)			
Grade 1	0	10(41.7%)	13(56.5%)			
Grade 2	7(33.3%)	12(50%)	0			
Grade 3	14(66.7%)	2(8.3%)	0			
Grade 4	0	0	0			
Lumbar lordosis (°)	56.22 ± 7.92	52.40 ± 7.16	54.18 ± 6.69	0.190	0.623	0.678
Operation time (min)	145.71 ± 21.11	129.17 ± 8.80	128.26 ± 7.75	0.0004*	0.0002*	0.9717
Blood loss (ml)	164.67 ± 17.99	144.58 ± 18.65	139.35 ± 19.79	0.002*	0.0001*	0.6096

*BMI* body mass index, *C-LSDI* China lumbar stiffness disability index P1 (PITF vs. TTL). P2 (PITF vs. EVO). P3 (TTL vs. EVO)

^*^The *p* values are from a one-way ANOVA

^†^The *p* values are from a chi-square test

^#^*p* < 0.05, Fisher’s exact test

### China lumbar stiffness disability index

**Fig. 4:**
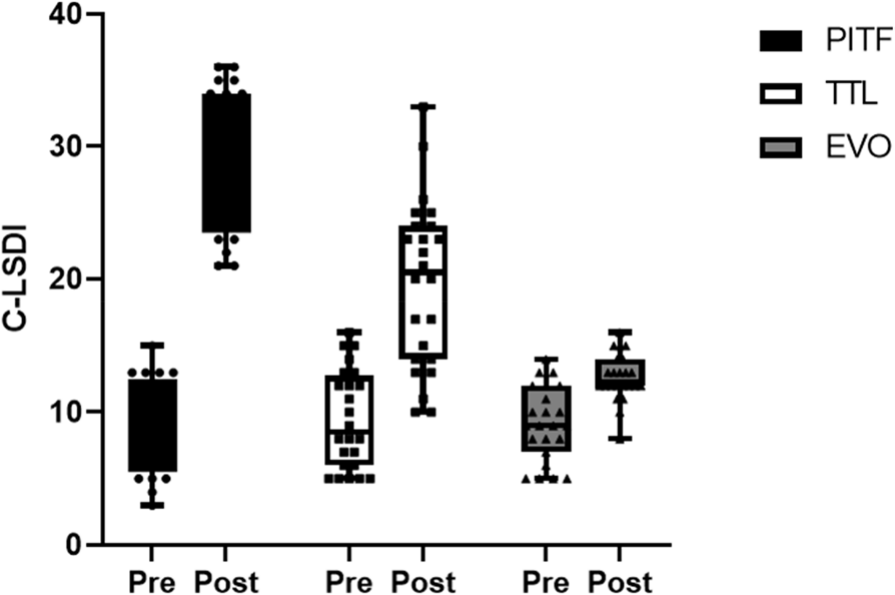
The C-LDI of pre- and post-operation among each group

**Fig. 5:**
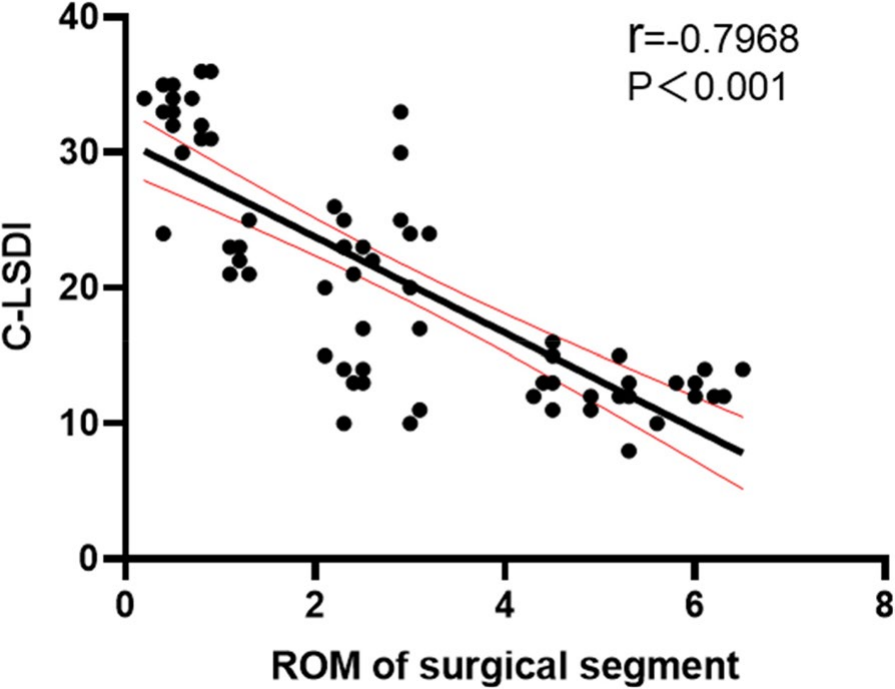
Correlation between surgical segmental mobility and C-LSDI

There was no significant difference in C-LDI scores between the three groups before surgery, and the scores in the PITF, TTL and EVO groups were 9.42 ± 3.52, 9.45 ± 3.69 and 9.13 ± 2.78, respectively (Table2 and Fig. 4). At the final follow-up, C-LSDI scores were significantly different among the three groups (*p* < 0.05). and the scores in the PITF, TTL and EVO groups were 29.76 ± 5.39, 19.71 ± 6.29 and 12.61 ± 1.78, respectively. The PITF group scored higher than the TTL group, the TTL group was slightly higher than the EVOL group, and the PITF group was significantly higher than the EVO group, and the C-LSDI score decreased with increasing surgical segment mobility, both of which were strong negative correlation (*r* = -0.7968, *P* < 0.001) (Fig. 5).

### ROM

#### ROM of surgical segment

**Fig. 6:**
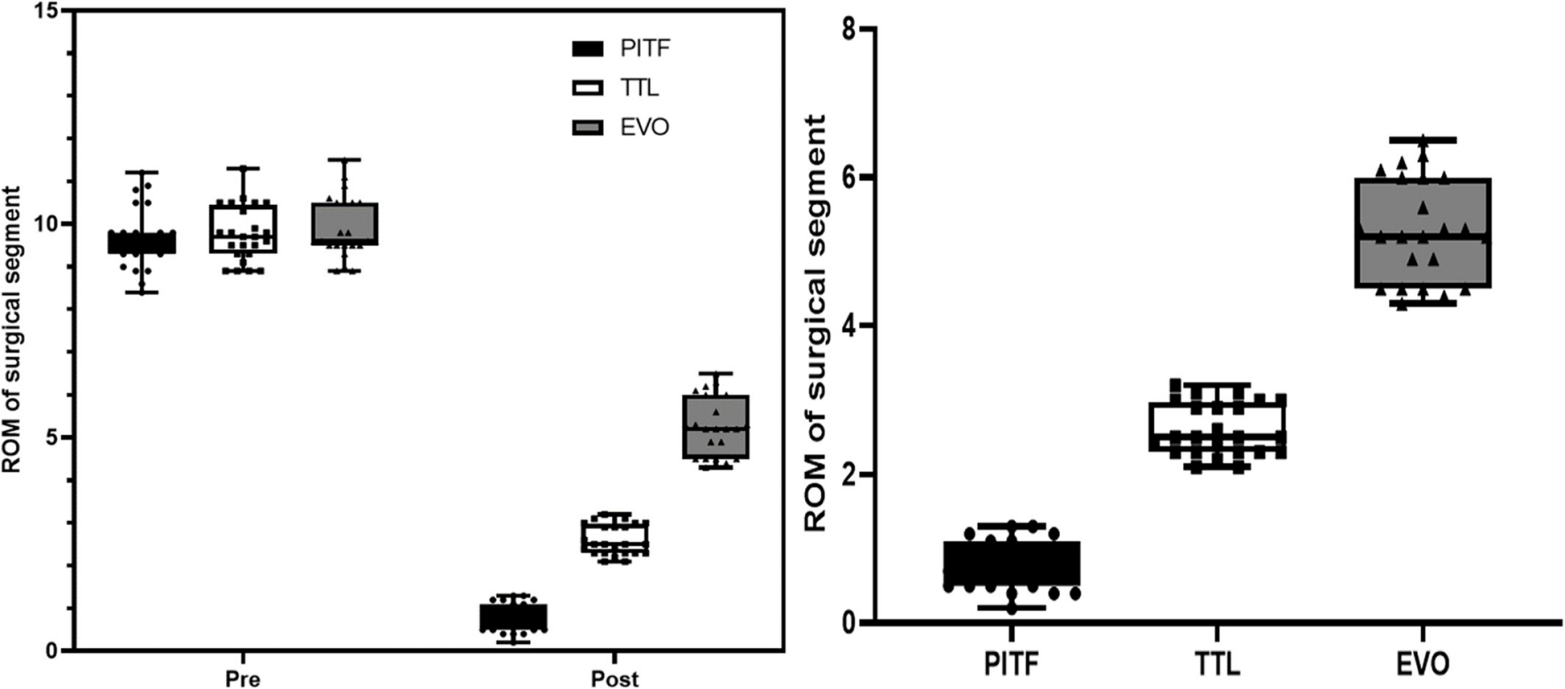
Pre- and post-operative comparison of ROM of surgical segment in three groups and inter-group comparison

There was no difference in fixed segment mobility between the PITF, TTL and EVO groups before surgery, which were (9.68 ± 0.75), (9.76 ± 0.65) and (9.90 ± 0.72), respectively. At the last follow-up, the ROM of surgical segment in the three groups was (0.77 ± 0.34), (2.60 ± 0.35) and (5.30 ± 0.68), respectively. All were significantly lower than the preoperative ones (*p *< 0.05), and between groups, the EVO group was significantly higher than the TTL group (*p *< 0.05) and the TTL group was significantly higher than the PITF group (*p* < 0.05) (Fig. 6).

#### ROM of upper adjacent segment

**Fig. 7:**
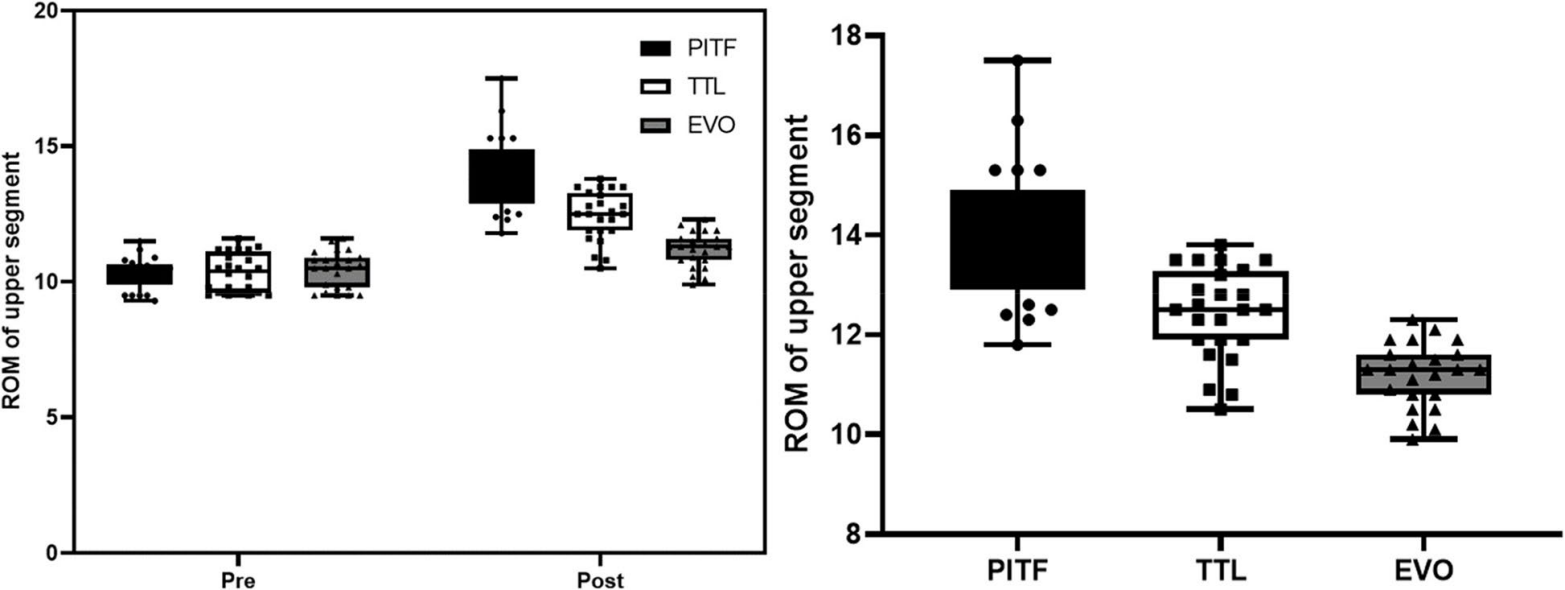
Pre- and post-operative comparison of ROM of upper adjacent segment in three groups and inter-group comparison

There was no difference in upper adjacent segment mobility between the PITF, TTL and EVO groups before surgery, which were (10.35 ± 0.59), (10.34 ± 0.71), (10.44 ± 0.68), respectively; at the last follow-up, upper adjacent segment mobility was (13.94 ± 1.43), (12.44 ± 0.92), (11.19 ± 0.65) in the three groups, respectively, which were significantly higher in all three groups than before surgery (*p* < 0.05) (Fig. 7).

#### Correlation of the ROM of surgical segment and upper neighboring segment

**Fig. 8:**
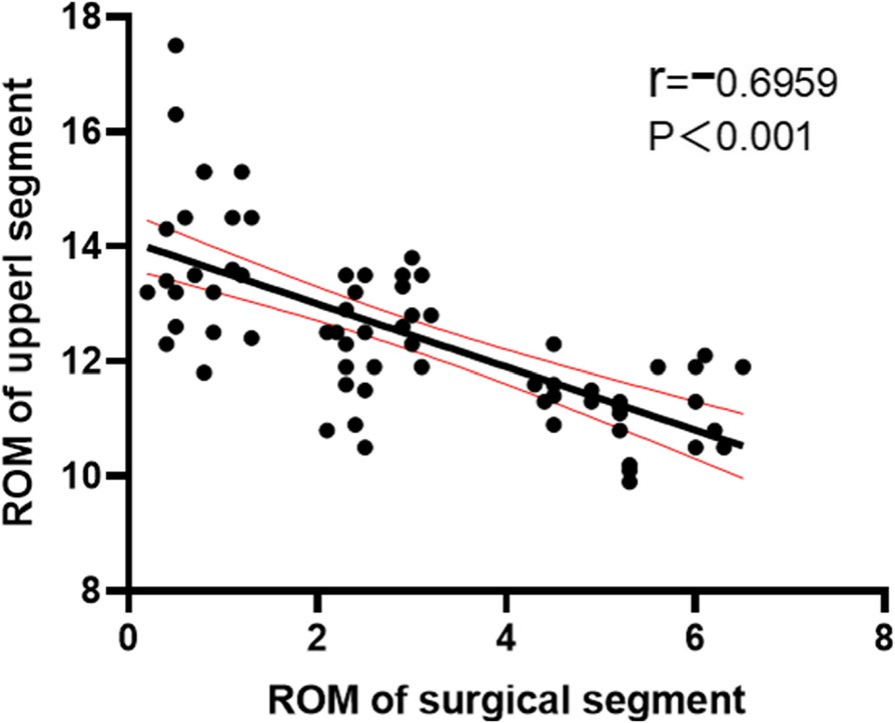
Correlation of ROM of surgical segment and upper adjacent segment

The ROM of upper adjacent segment activity increased with the decrease of ROM of surgical segment in all three groups, and there was a strong negative linear correlation between ROM of surgical segment and upper adjacent segment (*r *= -0.6959, *P* < 0.001) (Fig. 8).

#### Correlation of ROM and Goutallier classification

**Fig. 9:**
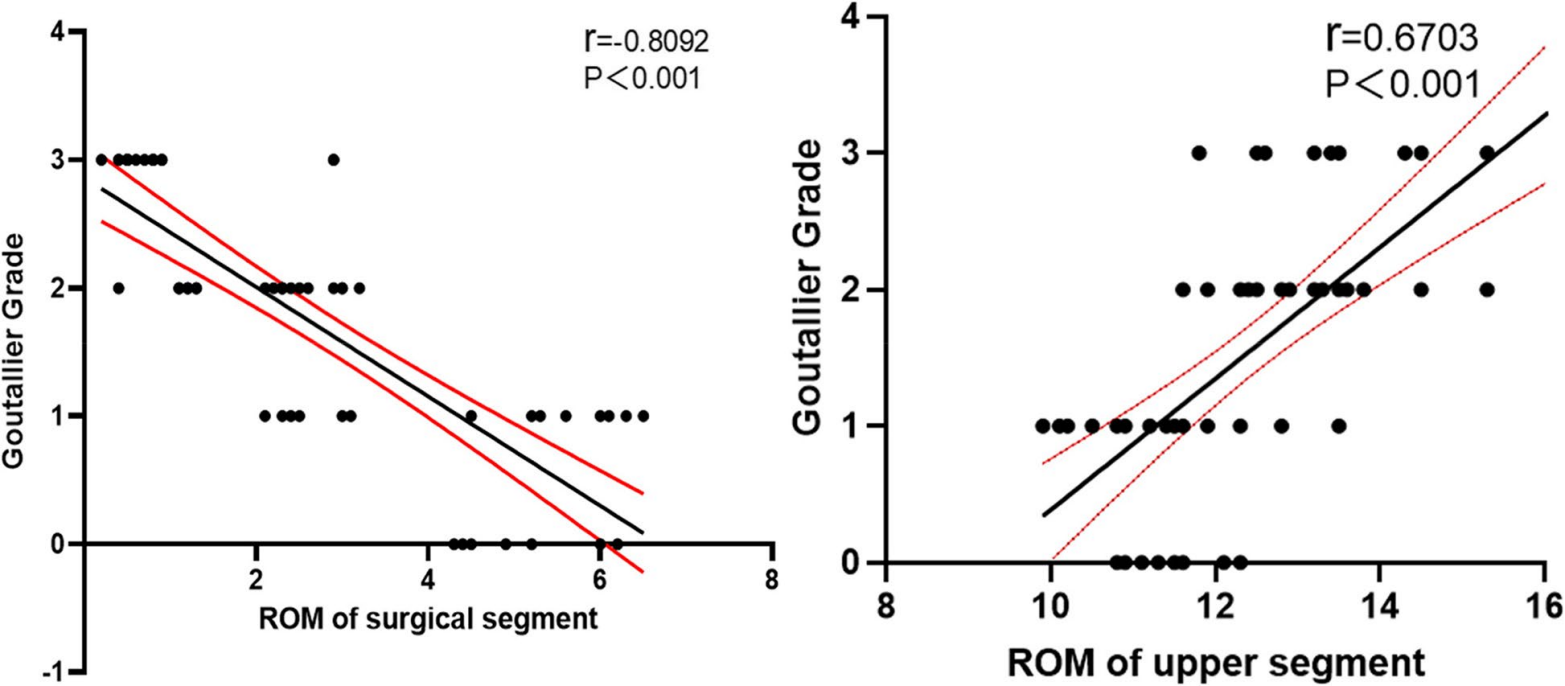
Correlation of ROM and Goutallier classification

**Fig. 10:**
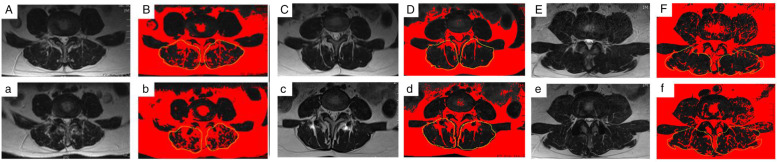
**A**. MRI T2 Axis map: The preoperative area of paraspinal muscles and fat of UAS in the PITF group, the Goutallier grade is 1. a. MRI T2 Axis map: The final-follow area of paraspinal muscles and fat of UAS in the PITF group, the Goutallier grade is 3. **C**. MRI T2 Axis map: The preoperative area of paraspinal muscles and fat of UAS in the Isobar TTL group, the Goutallier grade is 1. c. MRI T2 Axis map: The final-follow area of paraspinal muscles and fat of UAS in the Isobar TTL group, the Goutallier grade is 2. E. MRI T2 Axis map: The preoperative area of paraspinal muscles and fat of UAS in the Isobar EVO group, the Goutallier grade is 0. c. MRI T2 Axis map: The final-follow area of paraspinal muscles and fat of UAS in the Isobar EVO group, the Goutallier grade is 0. **B**, b, **D**, d, **F**, f. The periphery of the paraspinal muscles on the image was outlined using yellow line, and the red region of the muscular compartment represents fat. (MRI image processing by Image J software)

Spearman correlation analysis of surgical segment mobility was significantly linearly negatively correlated with upper adjacent segment Goutallier classification (*r* = -0.8092, *P* < 0.001), while upper adjacent segment mobility was significantly negatively correlated with Goutallier classification was strongly linearly positively correlated (*r* = 0.6703, *P* < 0.001) (Fig. 9 left). Correlation between surgical segment mobility and Gourallier classification in three groups, typical cases (Fig. 9 right). The Typical cases of each group were showed in Fig. 10.

## Discussion

### The relationship between mobility and low back stiffness

Dynamic stabilization technique can maintain a certain degree of mobility in the surgical segment of the lumbar spine to reduce the stiffness of the low back that is common after fusion, while limiting the abnormal movement of the surgical segment of the lumbar spine to reduce the compensatory activity of the adjacent segment, which can avoid the abnormal distribution of stress in the adjacent segment to some extent and reduce the incidence of ASD [36]. However, clinical research is currently divided on how much mobility should be retained and whether higher mobility is better for dynamic stabilization devices. The Isobar Dynamic Stabilization System has undergone five generations of design since its inception: Isolock (1993), Isobar TTL (1998), Aladyn (2002), Isobar Duo (2008), and Isobar EVO (2010), and has become a mature internal fixation device. The original design concept was based on Wolff's law to promote intervertebral fusion for fusion surgery. Since the 1980s, non-fusion stabilization systems such as the posterior lumbar interspinous spine bracing device, interspinous joint device, and posterior transforaminal dynamic internal fixation have been improved and evolved. Some clinicians have also used the Isobar dynamic stabilization system to improve clinical outcomes in non-fusion and hybrid technology [37–39]. This system provides spinal stability while preserving a certain degree of mobility of the operated segment, and the load transfer center of the surgical segment is close to the anterior-middle column of the spine, which results in less compressive stress than traditional rigid fixation devices. Given the mere mobility and subtle contour differences between EVO system and the TTL system, we believe it is more scientific to explore mobility through the EVO system and the TTL system for low back stiffness.

In this study, by comparing the C-LSDI before and after surgery in the PITF, TTL and EVO groups, we found significant differences in the C-LSDI of patients in the three groups, with the final follow-up score of the PITF group (29.76 ± 5.39), the TTL group (19.71 ± 6.29) and the EVO group (12.61 ± 1.78), indicating that the postoperative fusion group Patients had the most pronounced low back stiffness, and patients in the dynamic fixation group had lower low back stiffness than those in the rigid group (PITF group), and patients in the EVO group had the lowest low back stiffness scores. This implies that both non-fusion and fusion procedures reduce the mobility of the fixed segment, which causes an increase in the mobility of the superior adjacent segment. In a comparison between groups, the surgical segment mobility was significantly higher in the EVO and TTL groups than in the PITF group, and the upper adjacent segment mobility was lower than in the PITF group, indicating that the Isobar non-fusion procedure has an advantage in preserving surgical segment mobility. Subsequently, we performed a correlation analysis between mobility and low back stiffness and found a significant positive correlation between fixed segment mobility and C-LSDI (*r* = -0.7968, *P* < 0.001). This suggests that there is indeed a close relationship between mobility and low back stiffness, and that increasing the mobility of the surgical segment can effectively reduce the stiffness of the patient's low back after surgery, so it is believed that increasing the mobility of the dynamic stabilization system within a certain range may lead to better clinical outcomes.

### The relationship between mobility and fatty infiltration of paravertebral muscles

The paravertebral muscles are an important group of muscles for maintaining normal trunk posture and spinal stability, although it has been shown in anatomy and physiology that the multifidus muscle (MM) is the most important for maintaining lumbar joint stability, the erector spinae (ES), and the psoas (PS) and quadratus lumborum (QL) are also indispensable for maintaining lumbar spine stability [39]. Through our clinical observations and the results of most studies [23, 40, 41], fat infiltration in patients with lumbar degeneration is mainly found in MM and ES. Therefore, the overall level of fat infiltration in LM and ES was used in this study to reflect the level of fat infiltration in the paravertebral muscles. There is lots of evidence that there is a correlation between fatty infiltration of the paravertebral muscles and lumbar spine degeneration of multiple structures [42–44]. Faur et al. [45] noted a low correlation (*R* = 0.37) and significant association (ANOVA, P = 0.001, 95% CI: 2.07 to 8.14) between the grade of disc degeneration and lumbar multifidus fat atrophy and concluded that the degree of paravertebral muscle fat infiltration was mildly negatively correlated with the level of the disc. This suggests that intramuscular fat infiltration also bridges the gap between disc degeneration and paravertebral sarcopenia. Paravertebral muscle degeneration also progresses with age, including muscle size reduction and muscle fat infiltration [46]. However, there are no reports in the literature on the relationship between posterior lumbar internal fixation surgical segment mobility and upper adjacent segment mobility and fatty degeneration of the paravertebral muscles in adjacent segments. Because of the prevalence of ASD in the upper adjacent segment and the varying degrees of paravertebral muscle destruction in the fixed segment, the paravertebral muscles of the upper adjacent segment were chosen as the target muscle group for this study.

Current commonly used measures of paravertebral muscle degeneration include paravertebral muscle cross-sectional area (CSA) and fat infiltration grade. Among them, both CSA and fat infiltration grade are associated with low back pain, radiculopathy and spinal stenosis [47–50]. However, some reported results of CSA were not related to fatty infiltration of the paravertebral muscle [51, 52] and indicated that fatty infiltration is more reflective of paravertebral muscle degeneration compared to CSA [53, 54]. Kader et al. [55] and Parkkola et al. [56] found significant muscle atrophy of the paravertebral muscle even in the absence of reduced CSA, with the atrophied muscle being replaced by fatty and fibrous tissue. Therefore, the fatty infiltration of the paravertebral muscles is more sensitive to early paravertebral degeneration when analyzing whether paravertebral muscle degeneration is occurring. The commonly used grading of fat infiltration is the Goutallier grading, and Battaglia et al [57]. reported a significant correlation between the Goutallier classification and the rate of fat infiltration in the paravertebral muscles, which has also been shown to be a reliable grading system for fat infiltration in several studies [57, 58]. Therefore, this grading system was selected for the evaluation index of paravertebral muscle degeneration in this study. In addition, Lee et al [29]. found the most significant changes in the L4/5 and L3/4 segments by measuring paravertebral muscle CSA and fat infiltration rates at each segmental level in patients with lumbar paravertebral muscle degeneration. Jun et al [59]. found that the degree of fatty infiltration of the paravertebral muscles was negatively correlated with the lumbar lordosis by analyzing the imaging data of 50 elderly patients [60]. Accordingly, in order to minimize confounding factors, only patients with single-segment fixation at L4/5 and L5/S1 and no significant difference in lumbar lordosis at the last follow-up were included in this study.

The MRI results showed that the TTL group had a significantly lower Goutallier grade at the last follow-up than the PITF group, but because of the variability in intraoperative bleeding and operative time between the two (164.67 ± 17.99 mL vs. 144.58 ± 18.65 mL, 145.71 ± 21.11 min vs. 129.17 ± 8.80 min), in order to eliminate the effect of these factors, we therefore compared the EVO and TTL groups again. The results showed that the fixed segment mobility in the EVO group was significantly higher than that in the TTL group, the upper adjacent segment mobility in the EVO group was significantly lower than that in the TTL group, and the Goutallier grading of the paravertebral muscles in the upper adjacent segment in the EVO group was significantly lower than that in the TTL group. The Goutallier classification showed a significant negative correlation with the mobility of the fixed segment (*r* = -0.8092, *P* < 0.001) and a positive correlation with the mobility of the upper adjacent segment (*r* = 0.6703, *P* < 0.001), indicating that when the intervertebral mobility exceeds the normal level, the paravertebral muscles will show significant fatty infiltration, so increasing the mobility of the dynamic fixation device can reduce the fatty infiltration of the paravertebral muscles in the upper adjacent segment by reducing the mobility of the upper adjacent segment.

## Conclusion

Compared with decompression combined with rigid fusion, decompression combined with dynamic fixation for single-segment lumbar degenerative disease can significantly reduce postoperative low back stiffness. And a certain range of increased mobility of the dynamic stabilization device can effectively reduce the compensatory mobility of the upper adjacent segment and slow down the fatty infiltration of the paravertebral muscle in the adjacent segment.

### Limitation

In the current study, we established relatively strict inclusion and exclusion criteria, excluding as much as possible other factors that may induce paravertebral muscle degeneration, such as intervertebral discs, small joints, age, BMI, and anterior lumbar convexity angle; secondly, during the surgical operation, the intervertebral discs and synovial joints of the operated segments were protected as much as possible to reduce the interference with the biomechanical environment of the spinal unit; finally, for the fusion group we chose PITF because its surgical procedure is more similar to the Isobar system procedure, which better ensures the scientific validity of the controlled trial. However, there were also two main confounding factors in this study: first, the fusion group wore a postoperative lumbar brace for 3 months and the dynamic fixation group wore a brace for only 1 month after surgery, because the purpose of dynamic stabilization was to allow patients to exercise their lumbar back muscles earlier, so the early paravertebral muscle recovery would be more advantageous in the dynamic fixation group compared with the fusion group, which may also be one of the reasons why the paravertebral muscle fat infiltration grade was lower in the dynamic fixation group than in the fusion group at the final follow-up; second, there was a difference in the inclusion criteria, with lumbar spondylolisthesis limited to degree I in the dynamic fixation group and degree II in the fusion group, which may also explain the lower fat infiltration grade in the dynamic fixation group at the final follow-up.

## Data Availability

The datasets used and/or analyzed during the current study are available from the corresponding author on reasonable request.
